# Comparative Clinicopathological Features and p16 Expression in Squamous Cell Carcinoma and Adenocarcinoma of the Cervix: A Single-Center Retrospective Cohort Study in Saudi Arabia (2020–2024)

**DOI:** 10.3390/biomedicines14030686

**Published:** 2026-03-17

**Authors:** Emad Alqassim, Mashael J. Abu Alola, Ahmad Y. Alqassim, Asma Tulbah, Sarah Alawami, Abdulrahman Samman, Zainab Y. Azzouni, Amnah A. Shubayli, Arwa A. Al-Qahtani, Abdulrahman A. Alahmari, Fatimah Alhamlan, Ahmed A. Al-Qahtani

**Affiliations:** 1 Department of Anatomic Pathology, Pathology and Laboratory Medicine, King Faisal Specialist Hospital and Research Center, Riyadh 11211, Saudi Arabia; ealqassim@kfshrc.edu.sa (E.A.); tulbah@kfshrc.edu.sa (A.T.); salawami@kfshrc.edu.sa (S.A.); asamman1@kfshrc.edu.sa (A.S.); zsazzouni@kfshrc.edu.sa (Z.Y.A.); 2 Pathology Department, College of Medicine, Alfaisal University, Riyadh 11533, Saudi Arabia; 3 Department of Infection and Immunity, Research Centre, King Faisal Specialist Hospital and Research Centre, Riyadh 11211, Saudi Arabia; malsaeedi@kfshrc.edu.sa (M.J.A.A.); falhamlan@kfshrc.edu.sa (F.A.); 4 Department of Family and Community Medicine, College of Medicine, Jazan University, Jazan 45142, Saudi Arabia; aalqassim@jazanu.edu.sa; 5 Department of Obstetrics & Gynecology, King Faisal Specialist Hospital and Research Center, Riyadh 11211, Saudi Arabia; ashubayli@kfshrc.edu.sa; 6 Department of Family Medicine, College of Medicine, Imam Mohammad Ibn Saud Islamic University (IMSIU), Riyadh 11432, Saudi Arabia; arahalqahtani@imamu.edu.sa; 7 Department of Medical Laboratory, College of Applied Medical Sciences, Prince Sattam Bin Abdulaziz University, Alkharj 11942, Saudi Arabia; aa.alahmari@psau.edu.sa; 8 Department of Microbiology and Immunology, College of Medicine, Alfaisal University, Riyadh 11533, Saudi Arabia

**Keywords:** cancer, squamous cell carcinoma, adenocarcinoma, p16, survival analysis, retrospective study, HPV, clinical staging

## Abstract

**Background**: Cervical cancer remains a major global health burden, with squamous cell carcinoma (SCC) and adenocarcinoma (ADC) representing the two predominant histological subtypes. Comparative clinicopathological patterns between SCC and ADC in contemporary cohorts remain of interest, but inference is often limited by small single-center datasets. **Methods**: We conducted a retrospective single-center cohort analysis of cervical cancer patients treated between 2020 and 2024. Demographic, clinical, and pathological variables, including p16 immunohistochemistry, histological subtype, differentiation grade, FIGO stage, and survival status, were analyzed. Comparative analyses were performed using appropriate exact tests, and survival was assessed using Kaplan–Meier methods. **Results**: The cohort included 85 patients: 69 with squamous cell carcinoma and 16 with adenocarcinoma. Both subtypes demonstrated similarly high p16 positive rates (89.9% vs. 93.8%, *p* = 1.00). Menopausal status emerged as a distinguishing factor (*p* = 0.0047), with SCC patients more likely to be postmenopausal. SCC patients were older on average (52.16 vs. 48.2 years: *p* = 0.0131). Analyses involving p16 status were interpreted descriptively due to the very small number of p16-negative cases. Kaplan–Meier analysis revealed significant survival differences by clinical stage (log-rank *p* = 0.03), with high-stage patients showing progressive decline from 95% to 73% survival over five years, while low-stage patients maintained 100% survival. **Conclusions**: In this retrospective single-center cohort, SCC and ADC showed similar p16 positivity rates and clinical stage remained the most informative prognostic variable. Apparent subtype-related demographic differences and multivariable associations should be considered hypothesis-generating rather than definitive. Larger multicenter studies with standardized pathology and p16 assessment, direct HPV testing/genotyping, and more complete clinical and prevention-related data are needed before prognostic or clinical conclusions are drawn.

## 1. Introduction

Cervical cancer remains a major global health burden and an important cause of cancer-related morbidity and mortality among women. It is the fourth most common cancer in women worldwide, with approximately 660,000 new cases and 350,000 deaths in 2022 [1]. In Saudi Arabia, GLOBOCAN 2022 estimates indicate 332 new cases and 164 deaths from cervical cancer, confirming a persistent national disease burden despite lower absolute numbers than in many high-incidence settings [2]. Despite advances in HPV vaccination and screening programs implemented over the past decade, significant disparities in outcomes persist globally [3,4]. The two predominant histological subtypes, squamous cell carcinoma (SCC) and adenocarcinoma (ADC), account for the majority of cervical cancers [5,6].

The biological and clinical differences between SCC and ADC have been the subject of considerable research interest. While both subtypes are predominantly associated with high-risk HPV infection, with significant regional variation in genotype distribution beyond the traditional HPV 16 and 18 focus, emerging evidence suggests distinct epidemiological patterns, molecular characteristics, and clinical behaviors [7,8]. Adenocarcinoma has been associated with younger patient age at diagnosis, different anatomical distribution within the cervix, and potentially distinct treatment responses compared to squamous cell carcinoma. However, prognostic differences between histological subtypes remain controversial, with some studies demonstrating significantly worse survival for adenocarcinoma in both early-stage (HR = 1.39) and advanced-stage disease (HR = 1.21) [9], while other contemporary analyses report no significant histotype-specific survival differences. Contemporary treatment standardization following international guidelines minimizes therapeutic confounding variables, enhancing the validity of histological subtype comparisons [10]. During the cytology-based screening era, the relative proportion of adenocarcinoma increased in some high-income settings, primarily because cytological screening was more effective at detecting squamous precursors than glandular precursors [6,11,12] However, with the widespread implementation of HPV vaccination and HPV-based primary screening, both SCC and ADC incidence are expected to decline in vaccinated populations [13]. These evolving prevention strategies highlight the importance of interpreting histotype-specific patterns within their era-specific screening and vaccination context [14].

The role of HPV in cervical carcinogenesis is well-established, with p16 immunohistochemistry serving as a reliable surrogate marker for HPV-mediated transformation [15,16,17]. P16 overexpression, resulting from functional inactivation of the retinoblastoma pathway by HPV E7 oncoprotein, has emerged as both a diagnostic tool and favorable prognostic marker in cervical cancer, with P16-positive tumors demonstrating better treatment response and improved survival outcomes [18,19]. However, the comparative prevalence and prognostic significance of p16 positivity across different histological subtypes requires further elucidation.

Clinical staging remains the cornerstone of cervical cancer prognosis and treatment planning, with the International Federation of Gynecology and Obstetrics (FIGO) staging system serving as the primary framework for risk stratification [20,21]. Nevertheless, the identification of additional demographic, molecular, and clinical factors that influence disease presentation and outcomes could enhance prognostic accuracy and inform personalized treatment approaches [22,23].

Although previous studies have examined survival differences between SCC and ADC [3,14,24], the comparative prevalence and clinical relevance of molecular markers, particularly p16 positivity, across these histological subtypes in contemporary populations remains of interest. Furthermore, the prognostic significance of demographic factors, molecular markers, and their interactions with clinical staging requires further exploration in adequately powered studies to better understand patterns of disease severity and survival outcomes.

The primary aim of this study was to describe and compare demographic, clinical, and pathological features of squamous cell carcinoma and adenocarcinoma of the cervix in a contemporary single-center cohort. Secondary objectives included exploratory analyses of factors associated with advanced-stage presentation and a preliminary assessment of survival outcomes. Given the retrospective design and limited sample size, particularly the small number of adenocarcinoma cases and the imbalance in p16 subgroups, these analyses should be considered hypothesis-generating rather than definitive. This study was not designed to establish clinically actionable prognostic markers, but rather to provide descriptive comparative data and identify areas for further investigation in larger, multicenter cohorts with standardized pathology, HPV testing, and treatment data.

Interpreting histotype-specific patterns in this cohort requires understanding the local cervical cancer prevention context. In Saudi Arabia, HPV vaccines (Cervarix^®^ and Gardasil^®^) were approved in 2010, but were only incorporated into the national immunization schedule in 2022, targeting females aged 9–14 years through a school-based, publicly funded program; national uptake remains low at approximately 4% [25]. There is no organized national cervical cancer screening program; only opportunistic cytology-based (Pap smear) screening is available, primarily for married women, with estimated coverage of approximately 17–19% [26,27]. National clinical practice guidelines published in 2016 recommended HPV testing or cytology within three years of marriage until age 65, but a systematic population-based program has not been implemented [28]. More than 40% of cervical cancers in Saudi Arabia are diagnosed at advanced stages [27,28]. Consequently, the women in our cohort (diagnosed 2020–2024) were almost certainly unvaccinated and had limited systematic screening exposure, which should be considered when interpreting stage distribution and subtype patterns.

## 2. Materials and Methods

### 2.1. Study Design and Setting

We conducted a retrospective observational cohort study analyzing cervical cancer patients diagnosed and treated at King Faisal Specialist Hospital & Research Centre (KFSHRC), Riyadh, Saudi Arabia, between January 2020 and December 2024. KFSHRC is a tertiary care academic medical center, serving as a major regional referral center for Saudi Arabia and the Middle East [29]. The institution maintains international accreditation standards including Magnet Recognition Program^®^ and HIMSS Stage 7 certification, ensuring standardized oncology care protocols and comprehensive data collection systems.

### 2.2. Study Population

The study included all patients with histologically confirmed cervical cancer diagnosed during the study period. Inclusion criteria comprised: (1) histologically confirmed primary cervical cancer; (2) complete clinical staging information; (3) available demographic and clinical data; and (4) confirmed histological subtype (squamous cell carcinoma or adenocarcinoma). Exclusion criteria included: (1) other histological subtypes (neuroendocrine tumors, sarcomas); (2) recurrent disease; (3) incomplete staging information; and (4) patients lost to follow-up within 30 days of diagnosis.

All eligible cases meeting inclusion criteria during the study period (2020–2024) were included to maximize statistical power and ensure comprehensive representation of the patient population.

### 2.3. Data Collection

Patient data were collected from medical records and included the following variables: demographic characteristics (age at diagnosis, body mass index [BMI], marital status, menopausal status), clinical parameters (FIGO stage, degree of differentiation), molecular markers (p16 immunohistochemistry status), histological subtype, and outcome measures (vital status at last follow-up). Clinical staging was performed according to the 2018 FIGO staging system, with stages categorized as low (I–II) versus high (III–IV) for analytical purposes.

Data quality was ensured through dual verification by two independent reviewers, with discrepancies resolved by consensus. Missing data patterns were assessed, and cases with >20% missing critical variables were excluded from analysis. Complete case analysis was performed for multivariable modeling, with sensitivity analyses conducted to assess the impact of missing data on primary outcomes.

### 2.4. P16 Immunohistochemistry

P16 immunohistochemistry was performed on formalin-fixed, paraffin-embedded tumor tissue at the time of initial histopathological diagnosis as part of routine clinical workup, in accordance with current WHO/IECC recommendations for the classification of HPV-associated cervical carcinomas [30]. Primary antibody (E6H4 clone, Ventana, Medical Systems Inc. Tucson, AZ, USA) was used at 1.0 μg/mL on VENTANA BenchMark ULTRA, Ventana, Tucson, AZ, USA. Quality control measures included positive and negative controls with each batch. P16 positivity was defined as continuous strong nuclear and cytoplasmic staining of the basal cell layer with extension upward involving at least one-third of the epithelial thickness (“diffuse staining pattern”), consistent with established criteria for HPV-associated cervical cancer [30,31]. Focal or patchy nuclear staining patterns were considered negative.

### 2.5. Ethical Considerations

This study was conducted in accordance with the Declaration of Helsinki and approved by the King Faisal Specialist Hospital and Research Centre (KFSHRC) Institutional Review Board (IRB)/Research Ethics Committee (REC) (IRB # 2251677, approved 4 November 2025, valid for 12 months). Given the retrospective nature of this study using de-identified patient data from medical records, the KFSHRC IRB/REC granted a waiver for informed consent requirements in accordance with institutional guidelines for retrospective chart reviews using secondary data. Patient confidentiality and data privacy were maintained throughout the study period.

### 2.6. Statistical Analysis

Descriptive statistics were calculated for all variables, with continuous variables presented as means with standard deviations and categorical variables as frequencies and percentages. Comparative analysis between histological subtypes were performed using exact tests for categorical variables (Fisher’s exact test for binary variables and Fisher–Freeman–Halton exact tests for multi-level variables), selected a priori due to sparse cell counts in several categories, particularly for p16 status. All analyses involving p16 status were treated as descriptive and exploratory only, given the extreme imbalance between p16-positive and p16-negative cases, and no definitive inferential conclusions were drawn from these comparisons. Continuous variables were compared using the Mann–Whitney U test, as appropriate based on distributional assumptions. Survival analysis was performed using the Kaplan–Meier method, with survival curves compared using the log-rank test. Follow-up time was calculated from the date of diagnosis to the last clinical contact or death. All statistical analyses were performed using Python V3.12.3 (JupyterLab environment), with appropriate statistical packages. Complete case analysis was performed, and statistical significance was defined as *p* < 0.05.

## 3. Results

Of 90 patients initially screened with a cervical cancer diagnosis, 85 met the inclusion criteria, yielding a final study population of 85 patients (69 SCC, 16 ADC). Table 1 summarizes baseline patient and tumor characteristics stratified by p16 status. Most patients were ≥40 years, with obesity being the most common BMI category. The majority presented at advanced FIGO stages (III–IV) and with grade 2 or 3 histology. No statistically significant differences were observed between p16-positive and p16-negative tumors with respect to age, BMI, FIGO stage, or survival outcomes. Due to the small number of p16-negative cases, exact statistical tests were applied, and subcategory-specific inference was not performed. Although histologic grade and marital status showed nominal *p*-values below 0.05, these findings should be interpreted cautiously given sparse cell counts in the p16-negative group and were not supported by consistent distributional patterns across categories.

The comparative analysis between squamous cell carcinoma and adenocarcinoma revealed several notable differences in patient characteristics and clinical presentation (Table 2). However, menopausal status and age are only variables that emerged as a significant distinguishing factor, with squamous cell carcinoma patients more likely to be postmenopausal compared to adenocarcinoma patients. Age demonstrated a marginally significant trend, with squamous cell carcinoma patients being older on average compared to adenocarcinoma patients. This age difference aligns with the menopausal status findings and suggests potential age-related variations in histological subtype development rather than differences driven by tumor biology or disease stage.

**Figure 1:**
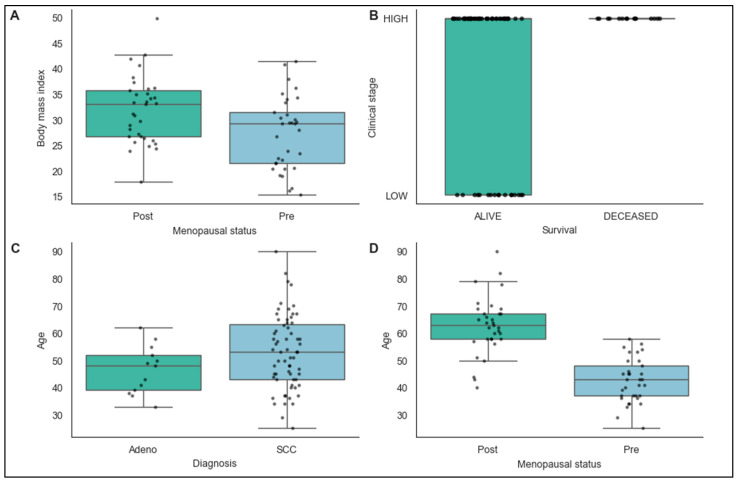
Associations between demographic, clinical, and pathological variables in cervical cancer patients. Multi-panel boxplot analysis examining relationships between key variables in the study cohort. (**A**): BMI distribution by menopausal status, showing significantly lower BMI in postmenopausal women (Mann–Whitney U test, *p* = 0.043). (**B**): Clinical stage distribution by survival status, with deceased patients presenting with more advanced disease compared with survivors (Mann–Whitney U test, *p* = 0.023). (**C**): Age distribution by histological subtype, demonstrating significantly older age among patients with squamous cell carcinoma compared with adenocarcinoma (Mann–Whitney U test, *p* = 0.013). (**D**): Age distribution by menopausal status, confirming the expected biological relationship between age and menopausal state (Mann–Whitney U test, *p* < 0.001). Box plots display the median (central line), interquartile range (box), and 1.5× IQR whiskers, with individual data points overlaid.

The boxplot analyses revealed several significant associations between key demographic, clinical, and pathological variables in the cervical cancer cohort (Figure 1). Postmenopausal women demonstrated a significantly lower BMI compared to premenopausal patients (Mann–Whitney U test, *p* = 0.043), suggesting potential metabolic differences associated with menopausal status. Most notably, deceased patients presented with more advanced clinical stage compared with living patients (Mann–Whitney U test, *p* = 0.023), confirming the prognostic importance of staging at diagnosis.

Age distribution varied by histological subtype, with squamous cell carcinoma patients being significantly older than adenocarcinoma patients (Mann–Whitney U test, *p* = 0.013). As expected, postmenopausal women were significantly older than premenopausal patients (Mann–Whitney U test, *p* < 0.001), validating the biological relationship between age and menopausal status.

**Figure 2:**
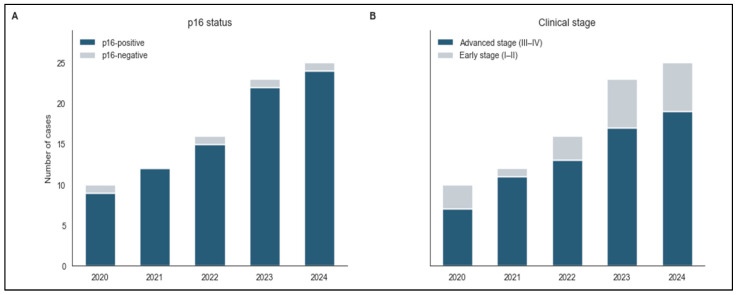
Distribution of p16 positivity and advanced-stage disease presentation across the study period (2020–2024). Bars represent the absolute number of p16-positive tumors (**A**) and advanced-stage cases (FIGO III–IV) (**B**) reported each year. Total case numbers were 10 (2020), 12 (2021), 16 (2022), 23 (2023), and 25 (2024). Corresponding p16-positive case counts were 9/10, 12/12, 15/16, 22/23, and 24/25, indicating that p16-negative tumors were rare across all years. Given the very small number of p16-negative cases, no formal temporal trend analysis was performed, and no inference regarding changes over time is implied. Year-to-year differences reflect variation in case accrual rather than systematic.

The temporal distribution of key clinical variables over the five-year study period from 2020 to 2024 (Figure 2). p16 positivity remained high throughout the study period, with modest year-to-year variation observed between 2020 and 2024, rising in 2020 to nearly complete positivity, then stabilizing at high levels through 2024. These observations may reflect improved diagnostic testing protocols, changes in patient population characteristics, or evolving epidemiological patterns of HPV-associated cervical cancers.

In contrast, the number of advanced-stage cases showed considerable year-to-year variation across the study period, fluctuating between 2020 and 2024 with no consistent pattern. Periods of higher case numbers were observed in some years, followed by lower counts in others, reflecting variability in annual case presentation. The lack of a consistent pattern in advanced-stage presentation suggests that factors influencing disease stage at diagnosis remain relatively stable over time, despite the consistently high prevalence of p16 positivity.

**Figure 3:**
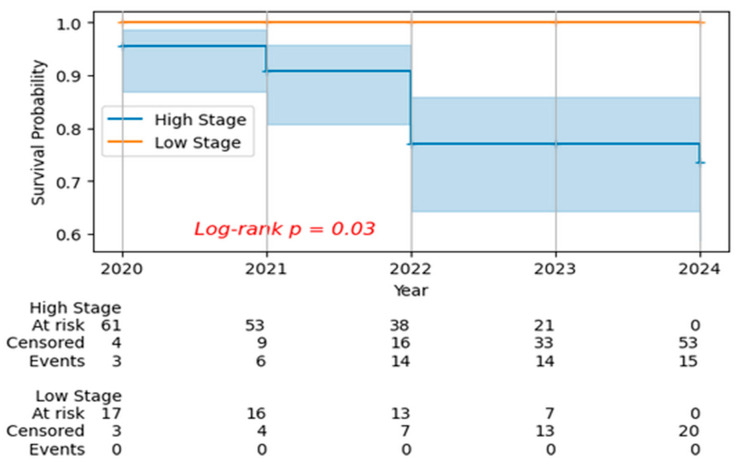
Kaplan–Meier Survival Curves by Clinical Stage in Cervical Cancer Patients (2020–2024). Time-to-event analysis comparing overall survival between low clinical stage (FIGO I–II, orange solid line) and high clinical stage (FIGO III–IV, blue solid line) cervical cancer patients over 60-month follow-up period. Low-stage patients maintained 100% survival probability throughout the entire observation period, while high-stage patients demonstrated progressive survival decline from 95% at baseline to 73% by 60 months (22% absolute survival reduction). The widening gap between curves demonstrates increasing mortality risk over time for advanced-stage disease. Log-rank test confirmed statistically significant difference between groups (*p* = 0.03). Number at risk tables are provided below the x-axis, censored observations are indicated by vertical tick marks, and shaded areas represent 95% confidence intervals. Median follow-up time was 3.0 years for both groups. These findings are consistent with clinical staging as a predictor of long-term outcomes and support the importance of early detection.

The Kaplan–Meier survival analysis demonstrated a significant difference in survival outcomes between patients with high and low clinical stage cervical cancer over the five-year study period (log-rank *p* = 0.03) (Figure 3). Patients with low clinical stage disease maintained excellent survival probability, remaining at 1.0 (100% survival) throughout the entire follow-up period from 2020 to 2024. In stark contrast, patients with high clinical stage disease showed progressive decline in survival probability over time, beginning at approximately 0.95 (95%) in 2020 and decreasing to 0.91 (91%) in 2021, followed by a more pronounced decline to 0.77 (77%) in both 2022 and 2023, and reaching 0.73 (73%) by 2024.

The median follow-up time was 3.0 years for both groups. This represents a 22-percentage-point absolute survival reduction (95% to 73%) or a 23% relative survival reduction over the five-year period for patients with advanced stage disease. Censoring events are indicated in the curves, and the numbers at risk at each year are displayed below the x-axis. The widening gap between survival curves over time is consistent with the well-established prognostic significance of clinical staging and supports its role as a predictor of long-term outcomes. However, survival differences were not adjusted for treatment effects, which may partially account for outcome variation.

## 4. Discussion

This retrospective single-center cohort study (N = 85) provides descriptive comparative data on the clinicopathological features of squamous cell carcinoma (SCC) and adenocarcinoma (ADC) of the cervix in a contemporary clinical setting. Overall, SCC and ADC showed broadly similar distributions for several clinical and pathological variables in this cohort, while some demographic differences were observed. However, the findings should be interpreted cautiously because of the limited sample size, particularly the small ADC subgroup (N = 16), the retrospective design, and the risk of model instability in multivariable analyses.

The observed differences in age and menopausal status between SCC and ADC were directionally consistent with prior epidemiological reports [32,33], with SCC patients tending to be older (52.16 vs. 48.2 years) and more often postmenopausal than ADC patients. However, both findings were of borderline statistical significance (age *p* = 0.057; menopausal status *p* = 0.050), and the limited number of ADC cases reduces statistical power for robust subtype comparisons. Accordingly, these results should be considered exploratory and hypothesis-generating rather than definitive evidence of histotype-specific demographic patterns in this population.

International literature has described historical screening-era shifts in cervical cancer histology, including a relative increase in the proportion of adenocarcinoma in some settings, particularly among younger women, partly attributed to lower sensitivity of cytology-based screening for glandular precursors compared with squamous precursors [5,34,35]. These reports provide relevant context for interpreting age differences between SCC and ADC. However, our dataset does not include individual screening histories, HPV vaccination status, or detailed population-level prevention context, and therefore cannot determine whether the observed age distribution in this cohort reflects screening patterns, vaccination effects, cohort effects, referral patterns, or underlying biological differences [36].

In addition, age and menopausal status are closely related variables, and their overlap should be considered when interpreting apparent differences between histological subtypes. The parallel direction of these findings may reflect the same underlying age structure rather than two independent signals. Larger multicenter cohorts with adequate representation of ADC and more complete covariate data (including screening and vaccination history) are needed to confirm whether these demographic differences persist after appropriate adjustment.

Taken together, the SCC–ADC differences observed in this study are best interpreted as descriptive comparative observations in a limited retrospective cohort. They are broadly compatible with prior epidemiological literature but do not support causal inferences regarding changing sexual behavior, HPV exposure patterns, latency, or histotype-specific pathophysiology, which were not directly measured in this study.

The similar p16 positivity rates observed in SCC and ADC in our cohort (89.9% vs. 93.8%) are broadly consistent with the established role of HPV-driven carcinogenesis across the major histological subtypes of cervical cancer and with prior reports showing high HPV prevalence in cervical adenocarcinoma as well as squamous cell carcinoma. However, p16 immunohistochemistry is a surrogate marker of HPV-mediated transformation and should not be interpreted as equivalent to direct HPV testing. Therefore, our findings support comparable p16 expression across SCC and ADC in this cohort, but they do not provide genotype-specific HPV information. The apparent association between p16 positivity and advanced clinical stage in our multivariable model (OR = 2.45, *p* = 0.047) should be interpreted with extreme caution and should not be considered a robust prognostic finding. This result is inconsistent with much of the published literature and is likely to reflect methodological limitations of the present study rather than a biologically meaningful effect. Most importantly, p16 status was highly imbalanced in our cohort, with only a very small p16-negative subgroup, making comparisons vulnerable to sparse-data bias, unstable effect estimates, and model overfitting. Several study features may have contributed to this unstable p16-stage association, including the retrospective single-center design, potential selection or referral bias, incomplete control of confounding, and possible temporal variation in p16 testing and interpretation practices over the study period. In addition, the observed increase in annual p16 positivity rates should not be overinterpreted as a reliable temporal trend because it is based on very small numbers of p16-negative cases and likely influenced by denominator variation and evolving testing patterns. It is also important to distinguish p16 status from HPV PCR-based HPV status when interpreting subgroup differences. A p16-negative tumor is not necessarily HPV-negative, and p16-negative cases may include HPV-associated tumors with absent or heterogeneous p16 expression, technical/pre-analytical staining variability, or interpretive variability, in addition to non-HPV-driven tumors. Accordingly, conclusions about “true HPV-negative” cancers cannot be drawn from p16 immunostaining alone in this dataset. Taken together, the p16 findings in this study are best viewed as descriptive and exploratory. They do not support clinical inference regarding p16 as an adverse prognostic marker in cervical cancer and should not be used to challenge established evidence without confirmation in larger, multicenter cohorts with standardized p16 assessment, direct HPV testing, and statistical methods appropriate for sparse-data settings.

The apparent association between marital status and advanced-stage presentation in the multivariable model (OR = 0.87, *p* = 0.021) should be interpreted cautiously and not considered a robust finding. Marital status is a multi-level social variable, and in a cohort of this size the corresponding estimates are vulnerable to sparse-data effects, category imbalance, and residual confounding. Although social determinants and social support may influence healthcare-seeking behavior, screening participation, and follow-up adherence, our dataset does not include the socioeconomic, behavioral, or screening-history variables needed to support mechanistic interpretation. Accordingly, marital status in this study should be regarded, at most, as an exploratory proxy variable rather than an independent prognostic determinant.

Similarly, the observed negative association between age and advanced stage should be interpreted with caution. Although this pattern appeared in multiple modeling approaches, the present dataset is small and susceptible to model instability and overfitting, and age is closely related to menopausal status, making independent interpretation difficult. We therefore avoid drawing strong conclusions regarding an age-related protective effect. Possible explanations such as differential healthcare contact, referral patterns, or cohort-specific factors remain speculative and cannot be evaluated with the available data. Larger cohorts with more complete clinical and prevention-related covariates are required to determine whether this association is reproducible.

The reported temporal increase in p16 positivity over the study period should also be interpreted very cautiously. Because the p16-negative subgroup was extremely small, yearly p16 positivity rates are highly sensitive to denominator variation and to the distribution of a very small number of p16-negative cases across years. As a result, the apparent trend may reflect random fluctuation and/or changes in testing and interpretation practices rather than a true temporal change in disease biology or epidemiology. For this reason, we consider the p16 temporal analysis descriptive only and do not regard it as evidence of improved diagnostic performance, diagnostic “maturity,” or changing population-level disease patterns.

More broadly, the combination of sparse p16-negative cases, retrospective single-center design, and limited prevention-context variables (e.g., screening history, HPV vaccination status, HPV genotyping) constrains interpretation of the associations reported in this study. These findings are best viewed as hypothesis-generating observations that require validation in larger, multicenter cohorts with standardized p16 assessment and more complete covariate data before clinical or etiologic inferences are made.

Our survival analysis showed clear separation of outcomes by clinical stage, with better survival among patients with early-stage disease than among those with advanced-stage disease during follow-up. This finding is consistent with the established prognostic importance of stage at diagnosis in cervical cancer. However, survival estimates in this cohort should be interpreted cautiously given the limited sample size, relatively short and variable follow-up, and the retrospective single-center design.

We did not observe a statistically significant survival difference between SCC and ADC in this cohort. This should not be interpreted as evidence of equivalent prognosis between histological subtypes, but rather as an absence of detectable difference in a small dataset with limited power, particularly for ADC (N = 16). Differences reported in prior studies may reflect larger sample sizes, different case mix, treatment patterns, subtype composition within adenocarcinoma, and longer follow-up.

Accordingly, the present data do not support strong clinical inferences regarding histological subtype as an independent prognostic factor beyond stage. In this cohort, stage appeared to be the dominant determinant of outcome, but the study was not adequately powered to evaluate histotype-specific survival effects with precision. Treatment-related conclusions should therefore be avoided.

Given the retrospective design and the contradictory or unstable findings in some analyses (particularly p16- and model-based associations), the clinical implications of this study should be interpreted cautiously. The apparent association between p16 positivity and advanced stage should not be used in clinical practice and should be considered exploratory only, pending confirmation in larger multicenter cohorts with standardized p16 assessment, direct HPV testing, and statistical methods appropriate for sparse-data settings.

Similarly, the observed demographic differences between SCC and ADC (age and menopausal status) are directionally consistent with prior epidemiological literature but were of borderline statistical significance in this cohort and should be regarded as hypothesis-generating rather than definitive. These findings may be useful as descriptive context, but they do not justify age-stratified clinical recommendations based on this dataset alone.

The main contribution of this study is descriptive: it provides contemporary single-center comparative data on SCC and ADC and highlights important methodological challenges in small retrospective cohorts, including sparse-data bias, unstable multivariable estimates, and the risk of overinterpreting subgroup and temporal analyses. Future studies should use larger multicenter cohorts with standardized pathology and p16 protocols, detailed treatment data, HPV genotyping, screening history, and HPV vaccination status to enable more reliable comparative and prognostic analyses.

Finally, the findings related to p16 and age-stage associations should be viewed as cautionary examples of how methodological limitations can generate potentially spurious associations that deviate from established evidence. These results underscore the importance of careful interpretation, transparent reporting, and external validation before drawing biological or clinical conclusions

## 5. Strengths and Limitations

This study has several strengths. It includes a contemporary real-world cohort of cervical cancer patients treated at a tertiary referral center and provides comparative data on the two major histological subtypes (SCC and ADC) within a defined study period (2020–2024). Clinical staging, histological subtype classification, and p16 immunohistochemistry data were available for most cases, enabling descriptive clinicopathological comparisons and exploratory analyses of stage at presentation and survival. The study also applies multivariable and temporal analytical approaches within a clearly defined retrospective cohort framework.

However, the limitations are substantial and should be emphasized more clearly. First, this is a single-center retrospective study with a small overall sample (N = 85), including only 16 ADC cases, which limits statistical power, precision, and generalizability. Second, p16 status was highly imbalanced (>88% p16-positive; only 4 p16-negative cases among patients with valid p16 staining), making p16 subgroup comparisons highly vulnerable to sparse-data bias, unstable estimates, and overfitting in multivariable models. As a result, the reported association between p16 positivity and advanced stage should be considered exploratory and not suitable for clinical inference. Third, several statistical approaches (including multivariable regression, LASSO-based feature selection, and temporal trend analysis of yearly p16 positivity) are difficult to justify in a dataset of this size and structure, particularly when annual rates are driven by very small numbers and denominator variation. Fourth, the study lacks key covariates needed for robust interpretation, including treatment details, screening history, HPV vaccination status, direct HPV test results/genotyping, and other important prognostic variables (e.g., lymphovascular invasion, parametrial involvement), as well as longer and more uniform follow-up for survival analyses. Fifth, the manuscript provides limited prevention-context information (screening and HPV vaccination practices in the study setting), which makes epidemiological interpretation of subtype patterns and stage distribution more difficult.

Overall, the findings should be interpreted as descriptive and hypothesis-generating. Larger multicenter studies with standardized pathology and p16 assessment, direct HPV testing, more complete clinical and prevention-related covariates, and statistical methods appropriate for sparse data are required before drawing prognostic or clinical conclusions.

## 6. Conclusions

This retrospective single-center cohort analysis provides descriptive comparative data on squamous cell carcinoma (SCC) and adenocarcinoma (ADC) of the cervix in a contemporary clinical setting. In this cohort, SCC and ADC showed similar p16 positivity rates, supporting the established role of HPV-related carcinogenesis across both major histological subtypes; however, p16 immunohistochemistry should be interpreted as a surrogate marker and not as equivalent to direct HPV testing. SCC patients in this cohort also tended to be older and more often postmenopausal than ADC patients, but these differences were of borderline statistical significance and should be considered exploratory.

Clinical stage remained the most informative prognostic variable in this study, with better survival observed in early-stage disease than in advanced-stage disease during follow-up. By contrast, the apparent associations observed for p16 status, marital status, and age in multivariable analyses should be interpreted with substantial caution because of the retrospective design, small sample size, limited ADC subgroup, incomplete covariate data, and sparse-data imbalance in p16 status (very few p16-negative cases), all of which increase the risk of unstable estimates and overinterpretation.

Overall, the findings of this study are descriptive and hypothesis-generating rather than definitive. Larger multicenter studies with standardized pathology and p16 assessment, direct HPV testing/genotyping, treatment data, screening and HPV vaccination history, and statistical methods appropriate for sparse data are needed to validate subtype-specific clinicopathological patterns and prognostic associations before clinical conclusions are drawn.

## Figures and Tables

**Table 1:** Baseline Patient and Tumor Characteristics.

Characteristic	Total (N = 81 *)	P16 Positive (N = 77)	P16 Negative (N = 4)	*p*-Value
**Age (years)**				**0.275**
<30	2 (2.5%)	2 (2.6%)	0 (0%)	
30–39	13 (16.0%)	13 (16.9%)	0 (0%)	
40–49	21 (25.9%)	20 (26.0%)	1 (25.0%)	
50–59	18 (22.2%)	18 (23.4%)	0 (0%)	
≥60	27 (33.4%)	24 (31.2%)	3 (75.0%)	
**BMI Categories**				**0.240**
Underweight (<18.5)	5 (6.2%)	5 (6.5%)	0 (0%)	
Normal (18.5–24.9)	15 (18.5%)	13 (16.9%)	2 (50%)	
Overweight (25–29.9)	22 (27.2%)	22 (28.6%)	0 (0%)	
Obese (≥30)	38 (46.9%)	36 (46.8%)	2 (50%)	
Missing	1 (1.2%)	1 (1.3%)	0 (0%)	
**FIGO Stage**				**0.306**
Stage I	4 (4.9%)	4 (5.2%)	0 (0%)	
Stage II	17 (21.0%)	17 (22.1%)	0 (0%)	
Stage III	39 (48.1%)	37 (48.1%)	2 (50%)	
Stage IV	21 (25.9%)	19 (24.7%)	2 (50%)	
**Histologic Grade**				**0.039**
Grade 1	6 (7.4%)	6 (7.8%)	0 (0%)	
Grade 2	49 (60.5%)	48 (62.3%)	1 (25.0%)	
Grade 3	22 (27.1%)	21 (27.3%)	1 (25.0%)	
Not reported	4 (4.9%)	2 (2.6%)	2 (50.0%)	
**Marital Status**				**0.027**
Married	49 (60.5%)	48 (62.3%)	1 (25%)	
Single	15 (18.5%)	15 (19.5%)	0 (0%)	
Divorced/Separated	6 (7.4%)	6 (7.8%)	0 (0%)	
Widowed	11 (13.6%)	8 (10.4%)	3 (75%)	

**Note:** * Out of 85 patients, 4 with Not reported P16 status and 1 labeled as Variable were excluded, leaving 81 patients (77 P16-positive and 4 P16-negative). For analysis, *p*-values were calculated using exact r × c tests (Fisher–Freeman–Halton; likelihood-ratio), due to sparse cell counts in the p16-negative group. Subcategory-specific inference was not performed.

**Table 2:** Comparison of Patient Characteristics and Clinical Features by Histological Subtype in Cervical Cancer (2020–2024).

Variable	Squamous Cell Carcinoma (N = 69)	Adenocarcinoma (N = 16)	*p*-Value
**P16 status**	Positive: 62 (89.9%) Negative: 3 (4.3%) Not reported: 3 (4.3%) Variable: 1 (1.4%)	Positive: 15 (93.8%) Negative: 1 (6.2%)	1.00
**Degree of differentiation**	Well: 6 (8.7%) Moderate: 41 (59.4%) Poor: 20 (29.0%) Not reported: 2 (2.9%)	Well: 0 (0.0%) Moderate: 12 (75.0%) Poor: 2 (12.5%) Not reported: 2 (12.5%)	0.107
**Clinical stage**	Early (I–II): 16 (23.2%) Advanced (III–IV): 53 (76.8%)	Early (I–II): 5 (31.2%) Advanced (III–IV): 11 (68.8%)	0.528
**Survival**	Alive: 50 (72.5%) Deceased: 11 (15.9%) Unknown: 8 (11.6%)	Alive: 10 (62.4%) Deceased: 3 (18.8%) Unknown: 3 (18.8%)	0.702
**Marital status**	Married: 42 (60.9%) Single: 13 (18.8%) Divorced: 5 (7.2%) Widowed: 9 (13.1%)	Married: 11 (68.8%) Single: 3 (18.8%) Divorced: 1 (6.2%) Widowed: 0 (0.0%) Unknown: 1 (6.2%)	0.148
**Menopausal status**	Positive: 36 (52.2%) Negative: 33 (47.8%)	Positive: 2 (12.5%) Negative: 14 (87.9%)	0.0047
**Age (years)**	Mean = 52.2	Mean = 48.2	0.0131
**BMI (kg/m^2^)**	Mean = 29.5	Mean = 28.9	0.722

**Note:** Categorical variables were compared using exact tests appropriate to variable structure (Fisher’s exact test for binary variables and Fisher–Freeman–Halton exact tests for multi-level variables), and continuous variables were compared using the Mann–Whitney U test.

## Data Availability

The datasets used and/or analyzed during the current study are available from the corresponding author on reasonable request.

## Abbreviations

The following abbreviations are used in this manuscript:

ADC/AC	Adenocarcinoma
HPV	Human Papillomavirus
FIGO	International Federation of Gynecology and Obstetrics
BMI	Body Mass Index
KFSHRC	King Faisal Specialist Hospital & Research Centre
